# A Four-Gene Prognostic Signature Based on the TEAD4 Differential Expression Predicts Overall Survival and Immune Microenvironment Estimation in Lung Adenocarcinoma

**DOI:** 10.3389/fphar.2022.874780

**Published:** 2022-05-04

**Authors:** Xiaoxia Gong, Ning Li, Chen Sun, Zhaoshui Li, Hao Xie

**Affiliations:** ^1^ School of Life Science and Technology, MOE Key Laboratory of Developmental Genes and Human Diseases, Southeast University, Nanjing, China; ^2^ Cardiovascular Department, Qingdao Hiser Hospital Affiliated to Qingdao University, Qingdao, China; ^3^ Hematology Department, Qingdao Hiser Hospital Affiliated to Qingdao University, Qingdao, China; ^4^ Qingdao Medical College, Qingdao University, Qingdao, China

**Keywords:** lung adenocarcinoma, TEAD4, prognostic signature, immune microenvironment estimation, biomarker

## Abstract

**Background:** TEA domain transcription factor 4 (TEAD4) is a member of the transcriptional enhancer factor (TEF) family of transcription factors, which is studied to be linked to the tumorigenesis and progression of various forms of cancers, including lung adenocarcinoma (LUAD). However, the specific function of this gene in the progression of LUAD remains to be explored.

**Method:** A total of 19 genes related to the Hippo pathway were analyzed to identify the significant genes involved in LUAD progression. The TCGA-LUAD data (n = 585) from public databases were mined, and the differentially expressed genes (DEGs) in patients with the differential level of *TEAD4* were identified. The univariate Cox regression, zero LASSO regression coefficients, and multivariate Cox regression were performed to identify the independent prognostic signatures. The immune microenvironment estimation in the two subgroups, including immune cell infiltration, HLA family genes, and immune checkpoint genes, was assessed. The Gene Set Enrichment Analysis (GSEA) and GO were conducted to analyze the functional enrichment of DEGs between the two risk groups. The potential drugs for the high-risk subtypes were forecasted *via* the mode of action (moa) module of the connectivity map (CMap) database.

**Results:** *TEAD4* was found to be significantly correlated with poor prognosis in LUAD-patients. A total of 102 DEGs in *TEAD4*-high vs. *TEAD4*-low groups were identified. Among these DEGs, four genes (*CPS1*, *ANLN*, *RHOV*, and *KRT6A*) were identified as the independent prognostic signature to conduct the Cox risk model. The immune microenvironment estimation indicated a strong relationship between the high *TEAD4* expression and immunotherapeutic resistance. The GSEA and GO showed that pathways, including cell cycle regulation, were enriched in the high-risk group, while immune response-related and metabolism biological processes were enriched in the low-risk group. Several small molecular perturbagens targeting *CFTR* or *PLA2G1B*, by the mode of action (moa) modules of the glucocorticoid receptor agonist, cyclooxygenase inhibitor, and NFkB pathway inhibitor, were predicted to be suited for the high-risk subtypes based on the high *TEAD4* expression.

**Conclusion:** The current study revealed *TEAD4* is an immune regulation–related predictor of prognosis and a novel therapeutic target for LUAD.

## Introduction

Lung cancer is one of the main causes of cancer-related death and is responsible for approximately 1.8 million deaths each year (Bray et al., 2018; Hoy et al., 2019). Approximately, 85% of these patients had non–small cell lung cancer (NSCLC), and the rest had small cell lung cancer (SCLC) (Sher et al., 2008; Byers and Rudin, 2015; Denisenko et al., 2018). Lung adenocarcinoma (LUAD) is the most common type of non–small cell lung cancer (NSCLC) and comprises approximately 40% of all lung cancer cases (Denisenko et al., 2018). Despite the improvement in current technology and techniques, the overall survival of LUAD has not been significantly improved, and only a fraction of patients benefited from therapies (Yamanashi et al., 2017; Schenk et al., 2021). Therefore, it is urgent to identify and explore more efficient therapeutic targets to further improve its prognosis.

The Hippo signaling pathway is evolutionarily conserved across higher order vertebrates, and by modulating target genes, it regulates multiple bioprocesses, including cell proliferation, survival, differentiation, and fate determination, as well as organ size and tissue homeostasis (Mohajan et al., 2021). Many of these roles are mediated by the transcriptional effectors Yes-associated protein (YAP) and its paralog transcriptional coactivator with the PDZ-binding motif (TAZ), which direct gene expression by control of a family of sequence-specific transcription factors called TEA DNA-binding proteins (TEAD1–4) that mediate proliferation and pro-survival genes (Dey et al., 2020; Masliantsev et al., 2021; Mohajan et al., 2021). Aberration of the Hippo pathway and YAP/TAZ-TEAD activity was recently shown to be linked to carcinogenesis in lung cancer (Huang et al., 2017; Liu et al., 2017; Gu et al., 2020). Overexpression of YAP/TAZ is associated with the development, progression, and poor prognosis of the disease (Mohajan et al., 2021). Therefore, the Hippo pathway is a novel tumor molecular biomarker and potential therapeutic target for LUAD. As one main component of the Hippo pathway, TEAD4 is a transcriptional enhancer–associated domain (TEAD) family protein (Pobbati and Hong, 2013) that plays biological roles by binding with DNA elements *via* its specific DNA-binding domains or through interaction with transcription coactivators (i.e., YAP/TAZ) by transactivation domains (Zhou et al., 2017; Gu et al., 2020). More recently, *TEAD4* has been demonstrated in tumorigenesis and cancer progression, including cancers of the breast (He et al., 2019; Wu Y et al., 2021), prostate (Chen CL et al., 2021), gastric (Shuai et al., 2020), bladder (Wu et al., 2019; Wang J et al., 2021), thyroid (Zhang et al., 2022), and lungs (Zhou et al., 2017; Gu et al., 2020; Hu et al., 2021; Yan et al., 2022). Previous studies have reported that *TEAD4* is upregulated in LUAD and is closely related to disease prognosis (Hu et al., 2021). However, the specific molecular mechanism of *TEAD4* regulation on the prognosis of LUAD is not understood.

The aim of the present study was to determine whether *TEAD4* could serve as a potential predictor of the prognosis of LUAD. We analyzed TCGA-LUAD samples with high and low *TEAD4* expressions, constructed a four-gene prognostic signature based on the *TEAD4* differential expression, and determined that *TEAD4* was an immune regulation-related predictor of prognosis for LUAD.

## Materials and Methods

### Data Acquisition

Gene expression sequencing data (HTSEQ-Counts and HTSEQ-FPKM) and the corresponding annotation of LUAD (n = 585) were acquired from the Genomic Data Commons (GDC) Portal (https://portal.gdc.cancer.gov/) of The Cancer Genome Atlas (TCGA) database (Tomczak et al., 2015). Excluding the data from the same patient, a total of 568 LUAD samples, including 58 normal (n_normal_ = 58) and 510 LUAD patients (n_LUAD_ = 510), were retained for the following differentially expressed gene (DEG) analysis. For the DEGs in *TEAD4*-high vs. *TEAD4*-low groups, the 510 samples of patients were divided into two subtypes according to the median TPM of *TEAD4*.

The clinical survival data (n = 738) and the phenotype data (n = 877) of TCGA-LUAD–matched patients were acquired from the GDC of the TCGA database. A total of 497 samples (n = 497), which contains both RNA-seq and survival data, were brought into the Cox model. For the nomogram analysis, a total of 383 samples (n = 383) were retained.

Gene Expression Omnibus (GEO) LUAD datasets were acquired and cleared up by the GEOquery R package. The validation sets of the Cox model were performed using GSE13213 (Tomida et al., 2009), GSE30219 (Rousseaux et al., 2013), and GSE31210 (Okayama et al., 2012), which contains 621 samples of LUAD.

### Identification of DEGs and the Enrichment Analysis

The DEGs with the threshold of fold change:2 and *p*-value < 0.05 were identified using HTSEQ-FPKM of TCGA-LUAD by the Deseq 2 R package (Love et al., 2014) and visualized by the ggplot2 R package. Gene Ontology (GO) (Ashburner et al., 2000) and Kyoto Encyclopedia of Genes and Genomes (KEGG) (Ogata et al., 1999) pathway enrichment analysis were conducted by clusterProfiler R package (Yu et al., 2012) and visualized by the ggplot2 R package. The Gene Set Enrichment Analysis (GSEA) (Subramanian et al., 2005) was performed by the WebGestalt online database (http://www.webgestalt.org/).

### Establishment and Validation of Prognostic Signature

Based on the TCGA-LUAD dataset (n = 497), univariate Cox regression, LASSO regression, and multivariate Cox regression analyses were used to screen the prognostic genes and establish the prognostic model. The survival R package was used to calculate the association between the expression of each DEG and overall survival (OS), and genes with *p*-value < 0.05 were retained for the following LASSO regression analysis. Glmnet and survival R package were used for the LASSO regression analysis to screen the significant variables in univariate Cox regression analysis. In order to obtain more accurate independent prognostic factors (prognostic characteristic genes), multivariate Cox regression analysis was used for the final screening. The risk score was calculated as follows: risk score = (exp-gene1*coef-gene1) + (exp-gene2*coef-gene2) + (exp-gene n*coef-gene n). Patients were divided into high- and low-risk groups based on the median of the risk score.

Time-dependent receiver operating characteristic (ROC) curves were used to assess survival predictions, and the Time ROCR package was used to calculate the area under the ROC curve (AUC) value to measure prognosis and predict accuracy. Survcomp R package was used for the C-index analysis. For the nomogram analysis, phenotype data (n = 382) were used and the clinical indexes, including age, gender, race, TNM staging, and stage, were brought into the COX regression analysis. For the external model construction, the risk score of the four independent prognostic signatures was calculated by the survival R package.

### TME Estimate Analysis

Stromal score, immune score, ESTIMATE score, and tumor purity score were calculated based on the mRNA expression (HTSEQ-Counts) by an estimate R package (Yoshihara et al., 2013). The significant static analysis was performed by the Wilcoxon rank-sum test.

The gene expression matrix data (HTSEQ-FPKM) were uploaded to CIBERSORT (Newman et al., 2015), and the immune cell infiltration matrix was obtained. ggplot2 R package was used to visualize the distribution of infiltration of 22 types of immune cells in each sample. The significant static analysis was performed by the Wilcoxon rank-sum test.

### Correlation Analysis of the Multigene

The correlation analysis of multiple genes was performed by Spearman’s correlation analysis and displayed by pheatmap R package.

### Chemotherapeutics Forecast

The chemotherapeutics forecast was performed using the mode of action (moa) module of the connectivity map (CMap, https://clue.io/command).

### Statistical Analysis

The statistical analysis was calculated *via* the Wilcoxon rank-sum test and unpaired t-text. All statistical tests were bilateral. All statistical tests and visualization were performed in R software (version 4.0.2).

## Results

### High *TEAD4* Expression Is Associated With Poor Prognosis in LUAD

**FIGURE 1:**
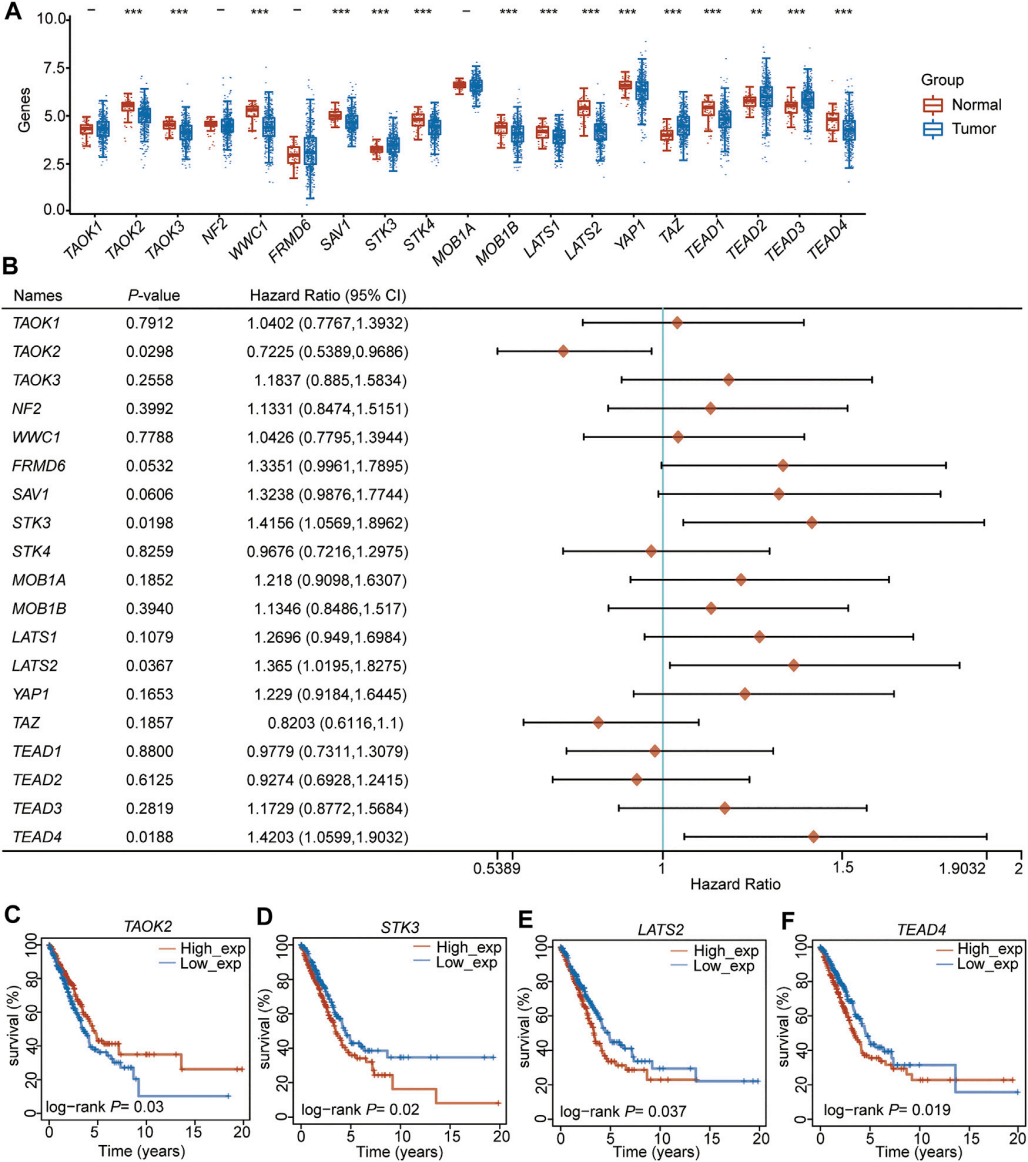
TEAD4 is downregulated in LUAD and associated with a poor prognosis. **(A)** Expression level (TPM) of the Hippo pathway-related genes in LUAD compared to adjacent normal samples. **(B)** Forest showing the prognosis of the Hippo pathway–related genes in LUAD. **(C–F)** Relationship between *TAOK2* **(C)**, *STK3* **(D)**, *LATS2* **(E)**, and *TEAD4* **(F)** expressions and OS in LUAD.

We selected 19 Hippo pathway-related genes (Wang et al., 2018) (Supplementary Table S1) and detected their expression levels in LUAD. The results showed that among these 19 genes, 11 (*TAOK2*, *TAOK3*, *WWC1*, *SAV1*, *STK4*, *MOB1B*, *LATS1*, *LATS2*, *TAP1*, *TEAD1*, and *TEAD4*) were downregulated in LUAD, while four (*STK3*, *TAZ*, *TEAD2*, and *TEAD3*) were upregulated in LUAD compared to adjacent normal samples (Figure 1A, Supplementary Table S2). To further evaluate the potential role of these genes in LUAD, the correlation between prognosis and these genes was analyzed, which showed that the expression of *TAOK2* was significantly associated with a superior prognosis (Figures 1B, C), while the expressions of *STK3*, *LATS2*, and *TEAD4* were associated with a poor outcome in LUAD (Figures 1B, D–F, Supplementary Table S3). These results suggest that the Hippo pathway plays an important role in the tumorigenesis and development of LUAD.

Univariate and multivariate regression analyses were performed for the four (*TAOK2*, *STK3*, *LATS2*, and *TEAD4*) prognosis-related genes, indicating that *STK3* (HR (HR.95L, HR.95H) =1.37 (0.96, 1.95), *p* = 0.077, Supplementary Table S4) and *TEAD4* (HR (HR.95L, HR.95H) =1.44 (1.17, 1.77), *p* = 0.0004, Supplementary Table S4) were independent prognostic signatures. Notably, *TEAD4* was found to have a prognostic value. Therefore, we focused on the analysis of *TEAD4*.

### Identification of DEGs Associated With *TEAD4* Differential Expression

**FIGURE 2:**
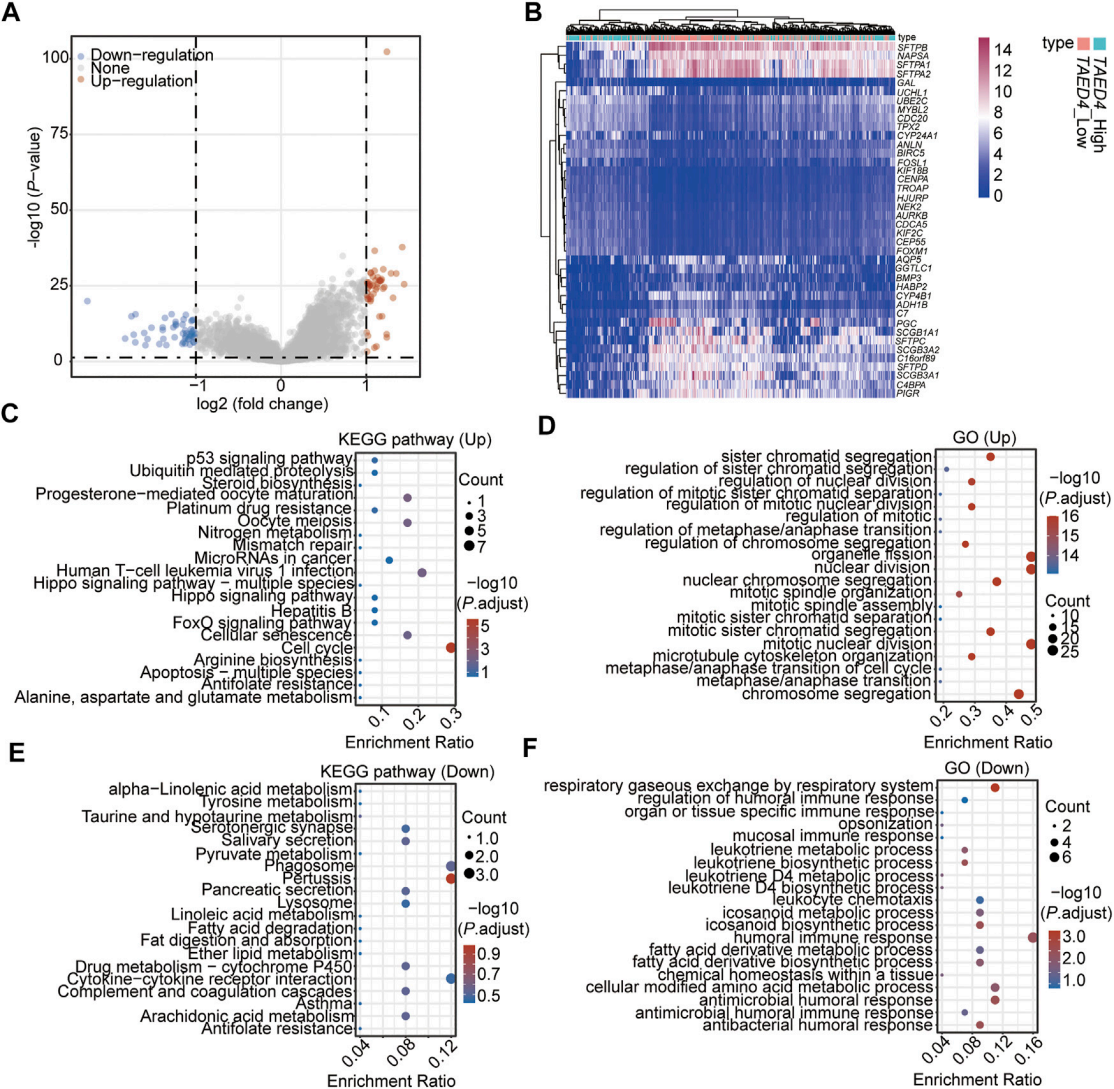
Identification of DEGs associated with the TEAD4 differential expression. **(A)** Volcano plot showing the DEGs in *TEAD4-*high vs. *TEAD4-*low groups. **(B)** Heatmap showing the top 20 up- and top 20 downregulated genes in *TEAD4-*high vs. *TEAD4-*low groups. **(C)** KEGG analysis of upregulated genes in the *TEAD4-*high group. **(D)** Enriched GO terms of upregulated genes in the *TEAD4-*high group. **(E)** KEGG analysis of downregulated genes in the *TEAD4-*high group. **(F)** Enriched GO terms of downregulated genes in the *TEAD4-*high group.

To explain the molecular mechanism of *TEAD4* in LUAD, the patients were divided into subgroups, *TEAD4*-high expression (*TEAD4*-high, n = 255) and *TEAD4*-low expression (*TEAD4*-low, n = 255), based on the median value, and the differentially expressed genes (DEGs) between the two subgroups were analyzed. A total of 102 DEGs (51 genes were up-regulated and 51 genes were downregulated) were identified in the *TEAD4*-high vs. *TEAD4*-low groups (Figures 2A,B, Supplementary Figure S1, Supplementary Table S5). The subsequent Kyoto Encyclopedia of Genes and Genomes (KEGG) and Gene Ontology (GO) results showed that upregulated genes in the *TEAD4*-high group belonged to pathways of the cell cycle, etc. (Figure 2C), and categories related to organelle fission, nuclear division, and chromosome segregation, etc. (Figure 2D). The downregulated genes in the *TEAD4*-high group belonged to pathways of pertussis, cytokine–cytokine receptor interaction, phagosome, etc. (Figure 2E), and were involved in bioprocesses of the humoral immune response, respiratory gaseous exchange by the respiratory system, and metabolic process, etc. (Figure 2F). These results demonstrate that the differential expression of this gene may lead to changes in the gene expression, which causes the dysregulation of cellular bioprocesses, including the cell division, immune response, and metabolic process.

### Establishment of a Prognostic Signature Based on the High *TEAD4* Expression in TCGA-LUAD

**FIGURE 3:**
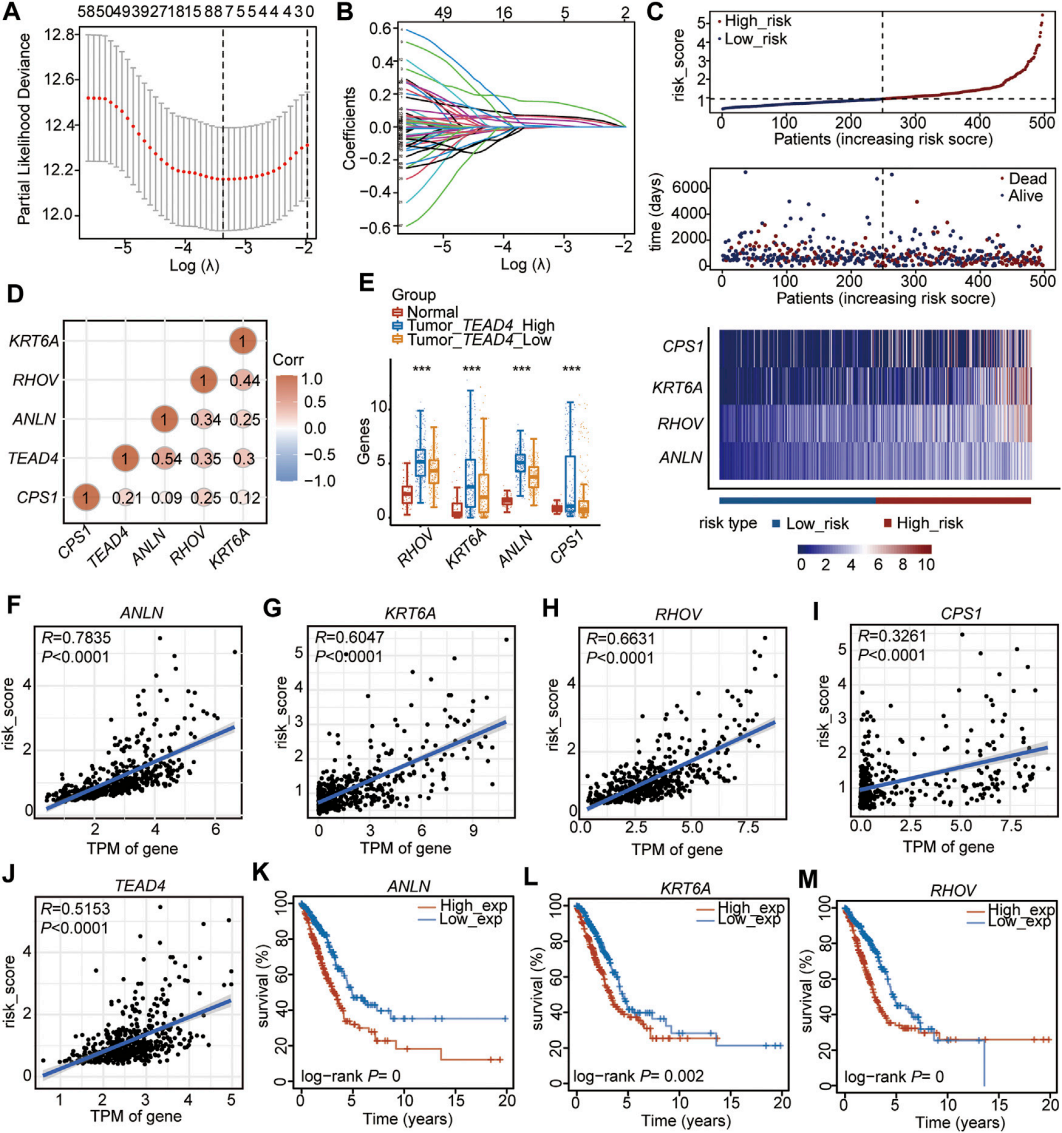
Establishment of the prognostic signature based on the high TEAD4 expression in TCGA-LUAD. **(A)** Stepwise Cox proportional risk regression model to screen the prognostic genes. **(B)** LASSO coefficient spectrum of prognostic gene screening. **(C)** Risk score distribution, survival status of patients, and heatmap of prognostic gene distribution in the training cohort. **(D)** Correlation analysis of prognostic genes and *TEAD4* in LUAD. The circle size represents significance. **(E)** TPM of the four prognostic genes in LUAD samples with *TEAD4-*high expressions, LUAD samples with *TEAD4-*low expressions, and the corresponding adjacent normal samples. The statistical significance was calculated *via* the Wilcoxon rank-sum test, ^***^ *p* < 0.001. **(F–J)** Correlation analysis between the prognostic genes and risk score. **(K–M)** Relationship between *ANLN* **(K)**, *KRT6A* **(L)**, and *RHOV* **(M)** expressions and OS in LUAD.

Among the 102 DEGs, 83 were found to be differentially expressed in LUAD tissues (n = 510) compared to adjacent normal tissues (n = 58) (Supplementary Table S6). These 83 DEGs were used to perform the univariate Cox regression analysis, and 74 genes (*p* < 0.05, Supplementary Table S7) were identified as prognostic genes. After suffering from zero LASSO regression coefficients, eight genes were identified to perform the multivariate Cox regression analysis (Figures 3A, B, Supplementary Table S8). Finally, four genes, carbamoyl phosphate synthetase 1 (*CPS1*), anillin actin-binding protein (*ANLN*), ras homolog family member V (*RHOV*), and keratin 6A (*KRT6A*), were identified as independent prognostic factors (Figure 3C, Supplementary Table S9). Based on the median of the risk score calculated by the expressions of these four genes, the 497 patients (patients without information on overall survival were excluded, n_497_ = n_510_-n_13_) were divided into two subtypes of high-risk and low-risk (Figure 3C, Supplementary Table S10). The high-risk subtype had significantly higher mortality rates than the low-risk group (Figure 3C). In addition, the expressions of the four independent prognostic signatures were higher in the high-risk group than in the low-risk subtype (Figure 3C), and the expression of each gene was positively correlated with the others (Figure 3D). Meanwhile, we found that these four signatures were positively correlated with the *TEAD4* expression (Figure 3D). These genes, which were all upregulated in LUAD, were highly expressed in the *TEAD4*-high subgroup (Figure 3E). The four genes and *TEAD4* were all significantly and positively associated with the risk score (Figures 3F–J). In addition, *TEAD4*, *ANLN*, *RHOV*, and *KRT6A* were all associated with the poor prognosis in LUAD (Figures 3K–M), indicating that these prognostic genes are of great significance for the evaluation of the LUAD outcome.

### Internal Validation of the Prognostic Signature

**FIGURE 4:**
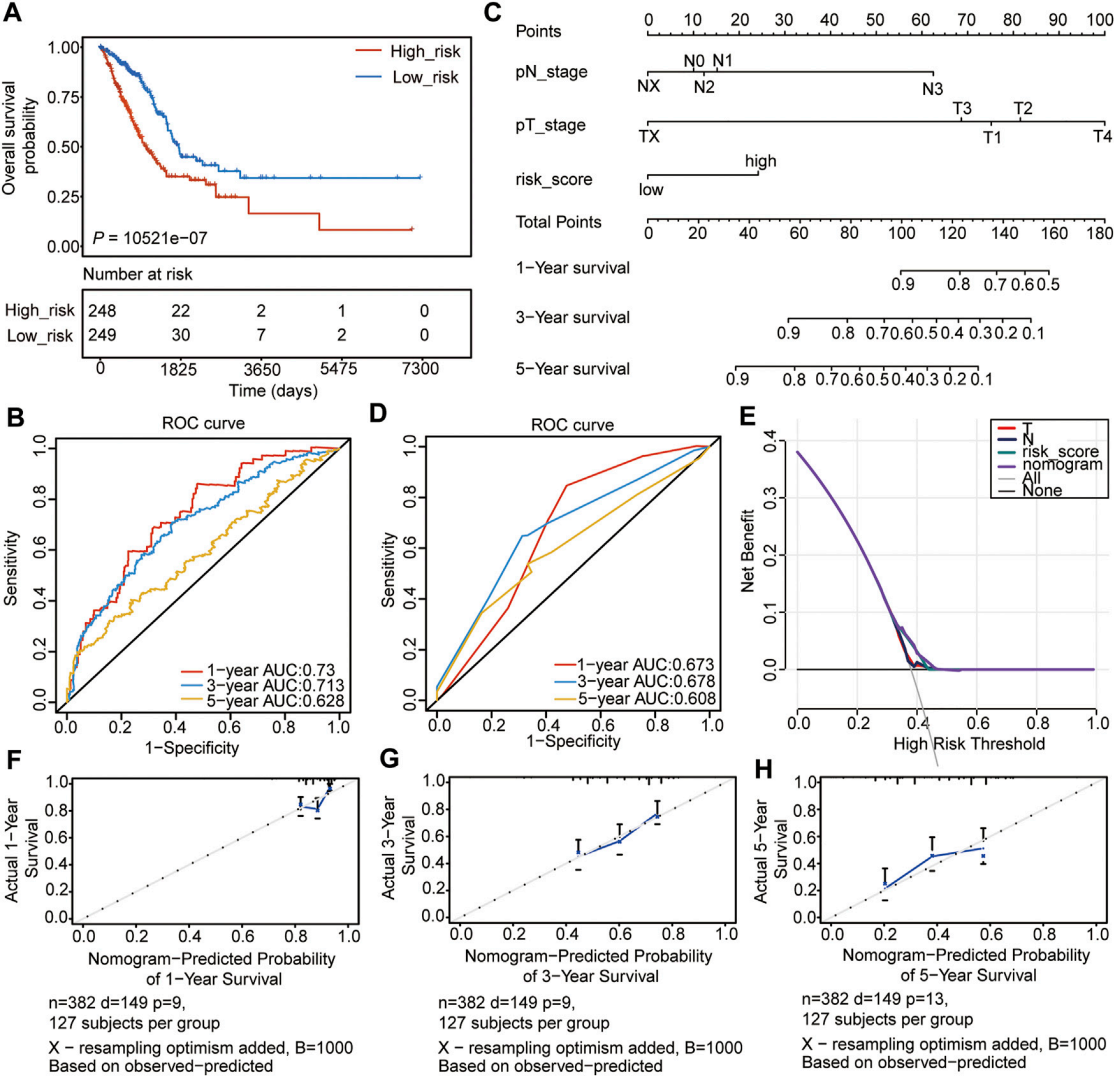
Internal validation of the prognostic signature. **(A)** OS of high- and low-risk groups. **(B)** ROC analyses of the model for 1-, 3-, and 5-years. **(C)** Nomogram to predict the prognosis of patients with LUAD. **(D)** ROC analyses of the nomogram model for 1-, 3-, and 5-years. **(E)** DCA result of the nomogram model. **(F–H)** The calibration analysis of the nomogram predicted the probability of 1-year survival **(F)**, 3-years survival **(G)**, and 1-year survival **(H)**.

We then evaluated the constructed risk model, and the overall survival (OS) analysis showed that the high-risk subtype had a poor prognosis (*p* =2.74e-07) (Figure 4A). The ROC curve was used to predict the prognosis at 1, 3, and 5 years, which showed that the prediction efficiency of the model was feasible (1-year AUC = 0.73; 3-year AUC = 0.713; 5-year AUC = 0.628) (Figure 4B). The concordance index (C-index) analysis also showed a consistent result (C-index = 0.6733, *p* = 2.704004e-15). Thereafter, the nomogram was constructed, and the clinical indices (Table 1, n = 382, age, gender, race, NTM staging) were incorporated into the nomogram to predict the OS of patients. The clinical indices of pT_satging and pN_satging were retained for further analysis after Cox regression analysis (Figure 4C). The ROC curve and C-index were used to predict the nomogram model, which showed a feasible result (1-year AUC = 0.673; 3-year AUC = 0.678; 5-year AUC = 0.608, C-index = 0.6763, *p* = 4.9488e-11) (Figure 4D). Moreover, the results of decision curve analysis (DCA) and calibration analysis of the nomogram predicted probability and also suggested the accuracy of the Cox model (Figures 4E–H).

**TABLE 1:** Clinical index of LUAD patients used in the Cox model.

Characteristic	Level	Overall	High-risk	Low-risk
n (dead/alive)		382 (149/233)	194 (92/102)	188 (57/131)
Age, n (%)	≥65	213 (55.76%)	103 (53.09%)	110 (58.51%)
	<65	169 (44.24%)	91 (46.91%)	78 (41.49%)
Gender, n (%)	Male	170 (44.50%)	89 (45.87%)	81 (43.08%)
	Female	212 (55.50%)	105 (54.13%)	107 (56.92%)
N stage, n (%)	N0	249 (65.18%)	124 (63.91%)	125 (66.48%)
	N1	65 (17.01%)	35 (18.04%)	30 (15.96%)
	N2	56 (14.65%)	31 (15.97%)	25 (13.29%)
	N3	1 (0.26%)	1 (0.51%)	0 (0)
	NX	11 (2.90%)	3 (1.57%)	8 (4.27%)
M stage, n (%)	M0	241 (63.08%)	122 (62.88%)	119 (63.29%)
	M1	21 (5.49%)	10 (5.15%)	11 (5.85%)
	MX	120 (31.43%)	62 (31.97%)	58 (30.84%)
T stage, n (%)	T1	132 (34.55%)	65 (33.50%)	67 (35.63%)
	T2	196 (51.30%)	96 (49.48%)	100 (53.19%)
	T3	40 (10.47%)	24 (12.37%)	16 (8.51%)
	T4	13 (3.40%)	8 (4.12%)	5 (2.67%)
	TX	1 (0.28%)	1 (0.53%)	0 (0%)
Pathologic stage, n (%)	Stage I	209 (54.71%)	96 (49.48%)	113 (60.10%)
	Stage II	87 (22.77%)	55 (28.35%)	32 (17.02%)
	Stage III	59 (15.44%)	31 (15.97%)	28 (14.89%)
	Stage IV	21 (5.49%)	10 (5.15%)	11 (5.85%)
	N/A	6 (1.59%)	2 (1.05%)	4 (2.14)

### Validation of the Cox Risk Model With Internal and External Sets

**FIGURE 5:**
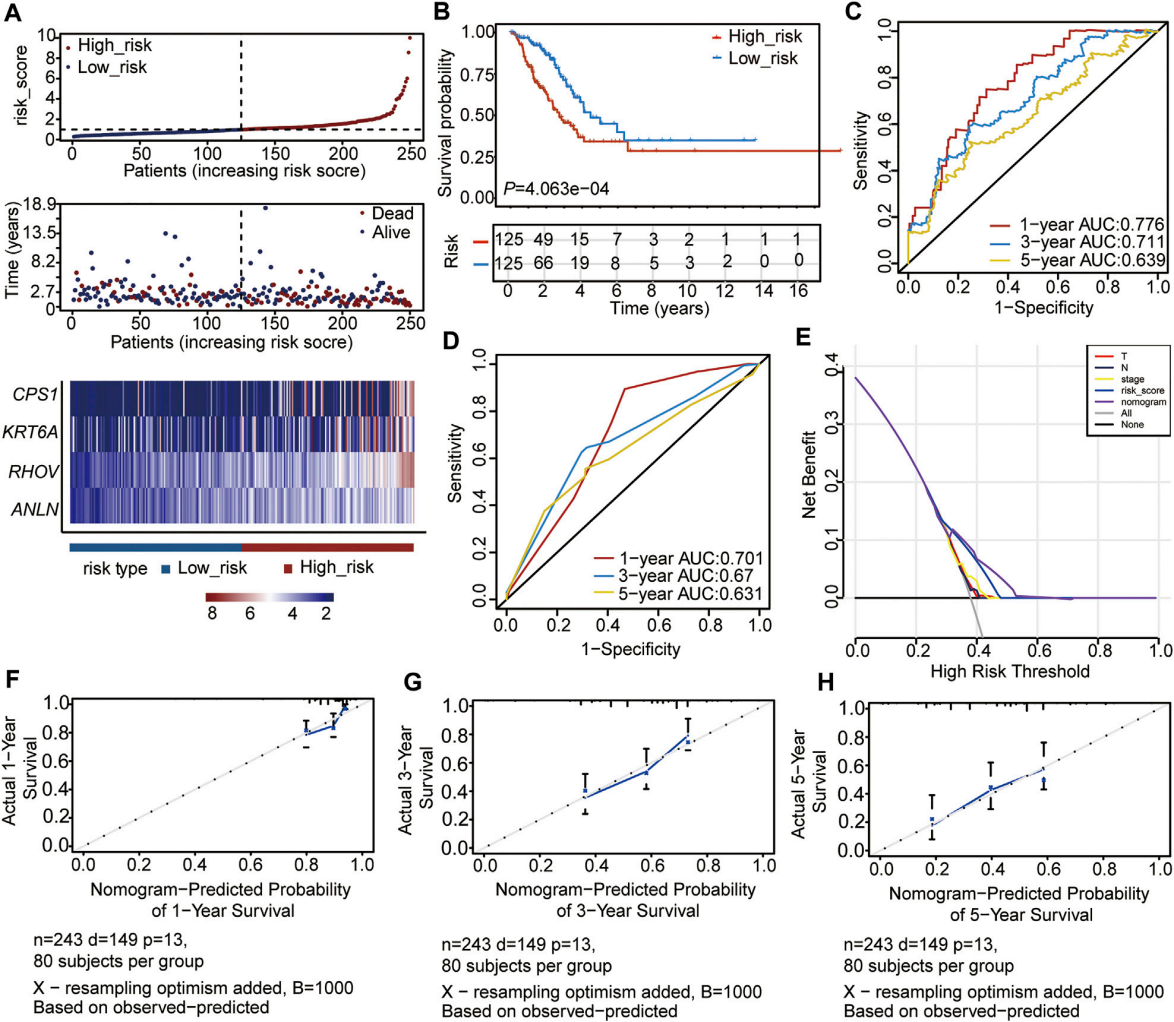
Validation of the Cox model with internal data. **(A)** Risk score distribution, survival status of patients, and heatmap of prognostic gene distribution in the validation set. **(B)** OS of high- and low-risk groups. **(C)** ROC curve of the Cox model for 1, 3, and 5 years. **(D)** ROC curve of the nomogram model for 1, 3, and 5 years. **(E)** DCA result of the nomogram model **(F–H)** Calibration analysis of the nomogram predicted the probability of 1-year survival **(F)**, 3-year survival **(G)**, and 5-year survival **(H)**.

To verify the accuracy of the four prognostic genes in predicting the outcome of LUAD, we selected 250 samples randomly from the 497 samples and re-reconstructed the Cox risk model using the four prognostic genes. We recalculated the risk score and divided the 250 samples into high- (n = 125) and low-risk (n = 125) subgroups, according to the median of the risk score (Figure 5A, Supplementary Table S11). Consistent with the previous results, the high-risk subtype had a higher mortality rate and higher expression levels of the four genes (Figure 5A). In addition, the high-risk subtype had a poor outcome compared to the low-risk subtype (*p* = 4.063e-04, Figure 5B). The ROC curve and C-index results also showed a feasible result (1-year AUC = 0.776; 3-year AUC = 0.711; 5-year AUC = 0.639, C-index = 0.6914, *p* = 1.719663e-11) (Figure 5C). Moreover, the clinical indices of pT_satging, pN_satging, and stage were retained for further analysis after Cox regression analysis (n = 243, Supplementary Table S12), and the corresponding ROC curve and C-index showed considerable results (1-year AUC = 0.701; 3-year AUC = 0.67; 5-year AUC = 0.631, C-index = 0.6932, *p* = 2.873223e-09) (Figure 5D). The DCA and calibration were also performed, and the results showed the high accuracy of the Cox model (Figures 5E–H).

To further validate the Cox model, GEO data sets, including GSE13213 (n = 117), GSE31210 (n = 226), and GSE30219 (n = 278), were acquired to construct the Cox model using the four-gene prognostic signature. In each validation set, patients were stratified into high- and low-risk groups, according to the median of the risk score (Table 2, Supplementary Table S13). The Kaplan–Meier survival analyses showed that patients in the high-risk subtype had significantly worse prognoses in all three validation sets (Supplementary Figures S2A–C). The ROC curve results showed that the AUCs of 1, 3, and 5 years in validation set 1 ranged from 0.71 to 0.95 (Supplementary Figure S2D), the AUCs of 1, 3, and 5 years in validation set 2 ranged from 0.722 to 0.925 (Supplementary Figure S2E), and the AUCs of 1, 3, and 5 years in validation set 3 ranged from 0.679 to 0.749 (Supplementary Figure S2F), which indicated the high accuracy of the model for evaluating prognosis. These demonstrate that the four-gene independent prognostic signature could be a promising factor for LUAD to predict the progression of tumor cells.

**TABLE 2:** Information of GEO data sets used in the validation of the Cox model.

Validation Set	GEO accession	Platform	Overall (1/0)	High-risk (1/0)	Low-risk (1/0)
Set 1	GSE13213	GPL6480	117 (49/68)	58 (33/25)	59 (16/43)
Set 2	GSE31210	GPL570	226 (35/191)	113 (29/84)	113 (6/107)
Set 3	GSE30219	GPL570	278 (188/90)	139 (111/28)	139 (77/62)

### Tumor Microenvironment Estimation of the Cox Model

**FIGURE 6:**
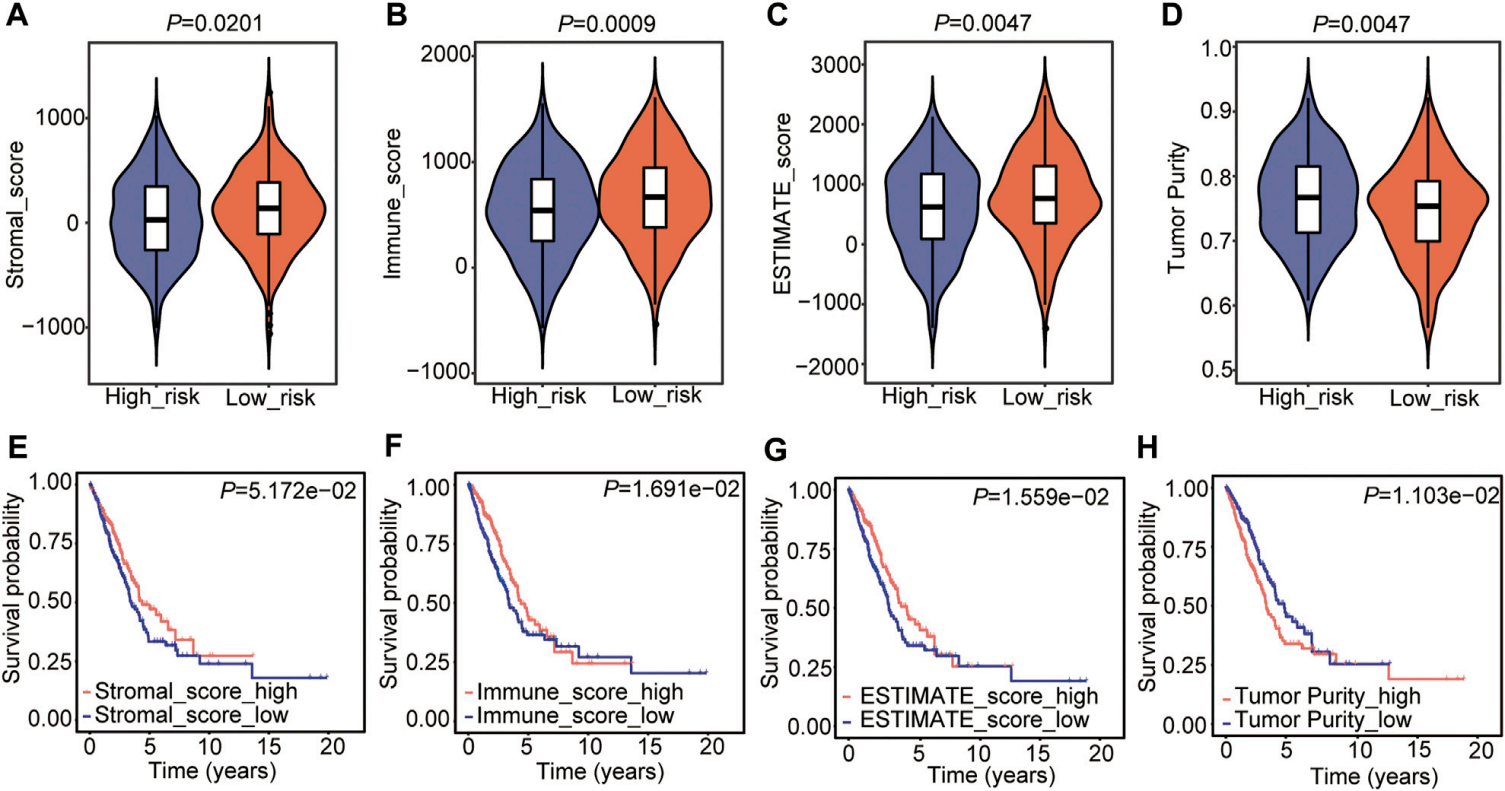
Tumor microenvironment estimation of the Cox model. **(A–D)** Comparison of the stromal score **(A)**, immune score **(B)**, estimate score **(C)**, and tumor purity **(D)** in the two risk groups. The statistical significance was calculated *via* the Wilcoxon rank-sum test. **(E–H)** The relationship between stromal score **(E)**, immune score **(F)**, estimate score **(G)**, and tumor purity **(H)** and OS in LUAD.

Subsequently, the tumor microenvironment (TME) in the two risk subtypes was analyzed, including the stromal score, immune score, ESTIMATE score, and tumor purity. The results showed that the high-risk subtype was featured with a lower stromal score, immune score, and ESTIMATE score (Figures 6A–C), as well as a higher tumor purity (Figure 6D). The subsequent relationship between the TME score and OS was analyzed and showed that high immune and ESTIMATE scores were associated with a good outcome in patients with LUAD (Figures 6F,G), while increased tumor purity was correlated with a poor prognosis (Figure 6H). The stromal score had no significant correlation with the prognosis in LUAD (Figure 6E).

### Infiltrating Proportion of Immune Cells in the Two Risk-Groups

**FIGURE 7:**
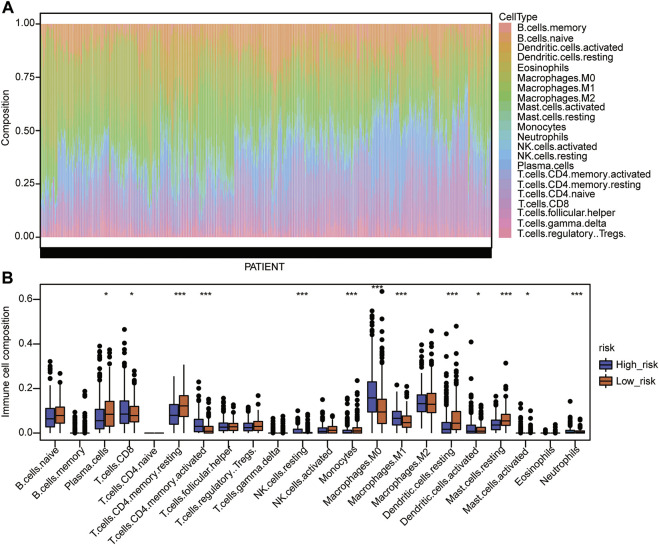
Immune cell infiltration analysis of the Cox model. **(A)** Landscape of immune cell infiltration in the TME of TCGA-LUAD. **(B)** Comparison of immune cell infiltration in the TME of the two risk groups. The statistical significance was calculated *via* the Wilcoxon rank-sum test, ^*^ *p* < 0.05, ^**^ *p* < 0.01, and ^***^ *p* < 0.001.

Immune cell infiltration was then analyzed, and the proportion of immune cell infiltration in the TME was first calculated by the CIBERSORT algorithm (Supplementary Table S14). The landscape of immune cells in the LUAD-TME showed great heterogeneity (Figure 7A). Among these, macrophages and T cells were the main groups (Figure 7A). Notably, the high-risk group had a lower proportion of plasma cells, resting memory CD4 T cells, monocytes, resting dendritic cells, and resting mast cells (Figure 7B). Meanwhile, the proportions of CD8 T cells, activated memory CD4 T cells, resting NK cells, M0 macrophages, M1 macrophages, activated dendritic cells, activated mast cells, and neutrophils were higher in the high-risk group (Figure 7B). These results suggest that the two risk groups had a distinct TME, which may alter the oncotherapeutic effect.

### HLA Family Gene Analysis of the Cox Model

**FIGURE 8:**
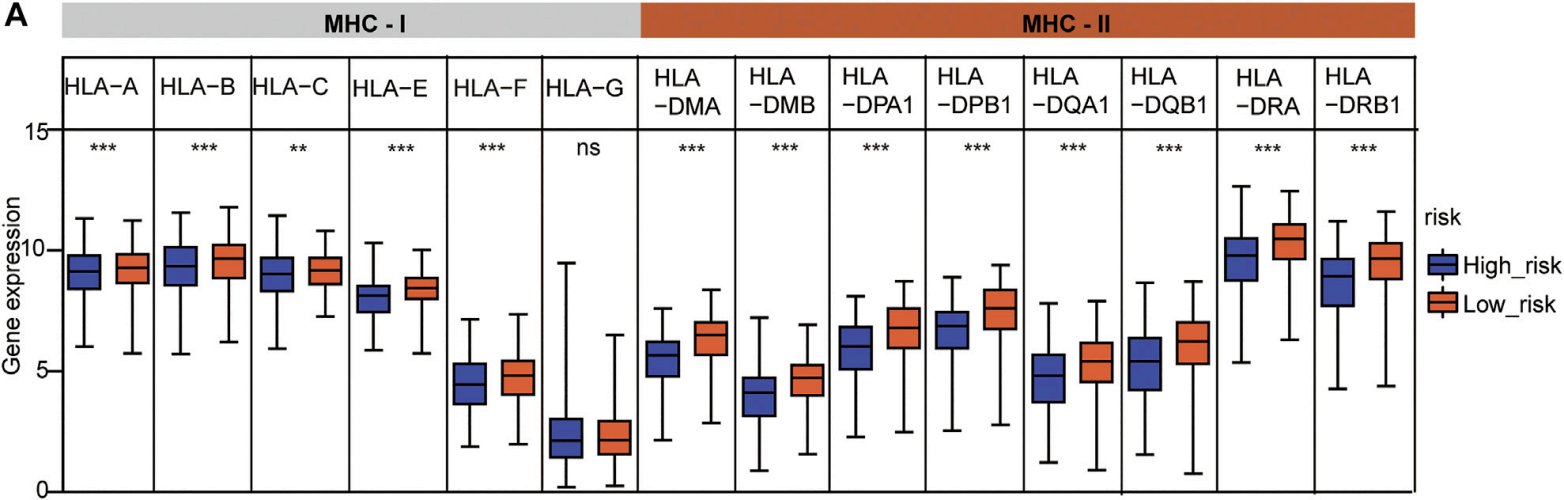
HLA gene expression analysis between the two risk subtypes. **(A)** HLA family expression analysis in the two risk groups. The statistical significance was calculated *via t*-test, **p < 0.01 and ***p < 0.001.

HLA family genes are the most complex and polymorphic genes which contain the most concentrated genes related to immune regulation that are involved in multiple diseases. We analyzed major histocompatibility complex, class I (MHC-I) and major histocompatibility complex, class II (MHC-II) expression between the two risks groups, and the two common HLA genes (Supplementary Table S1). The results showed that five of six MHC-I genes (*HLA-A*, *HLA-B*, *HLA-C*, *HLA-E*, and *HLA-F*) and all MHC-II genes were significantly under-expressed in the high-risk group (Figure 8A), indicating a feasible poor antitumor immune response in the high-risk group.

### Immune Checkpoint Gene Analysis in the Two Risk Subtypes

**FIGURE 9:**
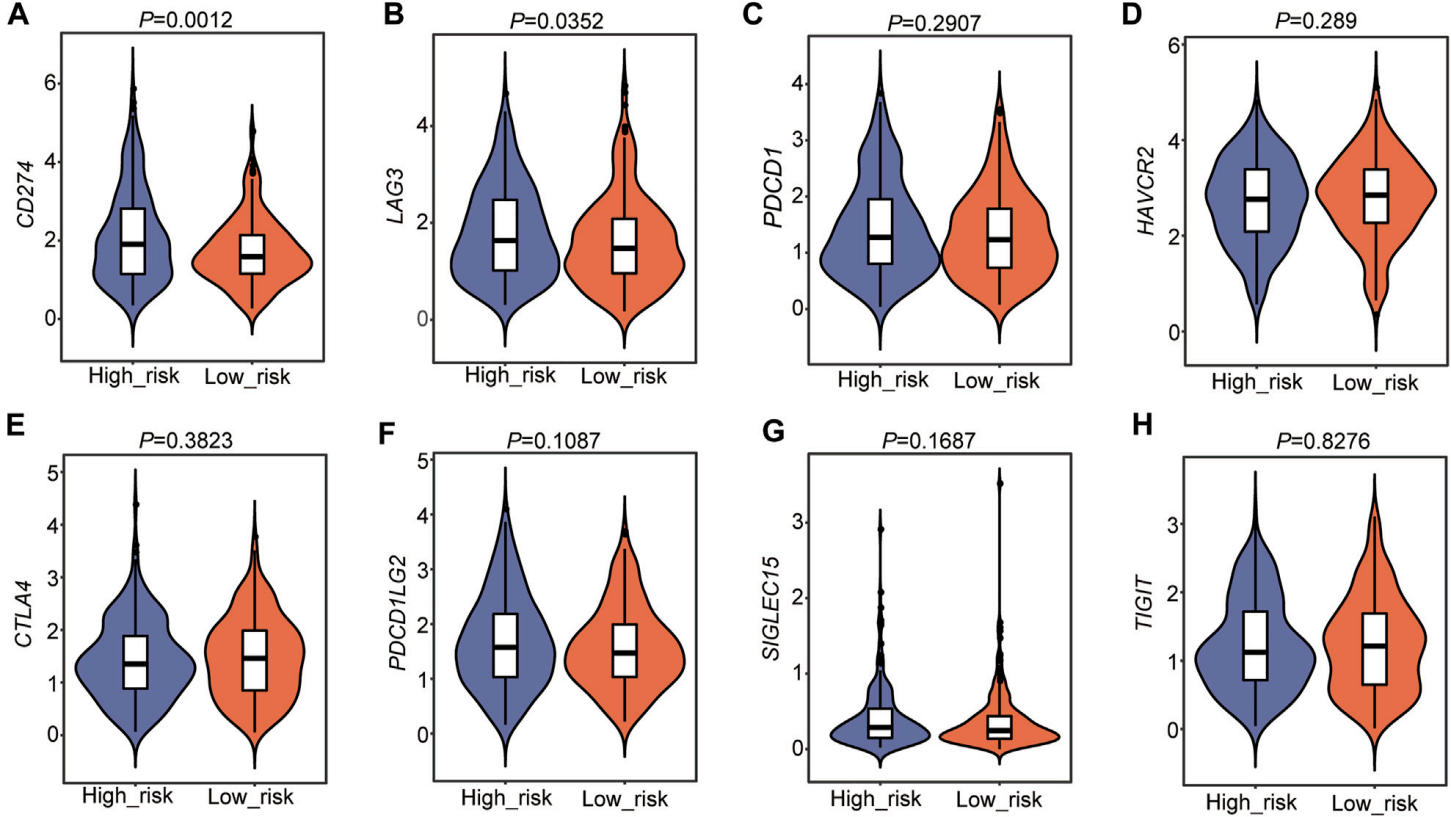
Immune checkpoint gene analysis in the two risk subtypes. **(A–H)** Immune checkpoint gene analysis in the two risk groups. The statistical significance was calculated *via* the Wilcoxon rank-sum test.

We then detected immune checkpoint genes, including *CD274* (*PD-L1*), *PDCD1* (*PD-1*), *LAG3* (CD223), *HAVCR2*, *CTLA4*, *PDCD1LG2*, *SIGLEC15*, and *TIGIT*, in the two risk subtypes (Supplementary Table S1). The results showed that the expression levels of *CD274* (*PD-L1*) and *LAG3* (CD223) were higher in the high-risk group than in the low-risk group (Figures 9A,B). Other genes were not different between the two groups (Figures 9C–H). These results suggested that a high *TEAD4* expression may predict immune checkpoint activity and reduce the immune checkpoint block (ICB) efficacy, thus promoting tumor cell survival and metastasis.

### Identification and Enrichment Analysis of DEGs Between the Two Risk Subtypes

**FIGURE 10:**
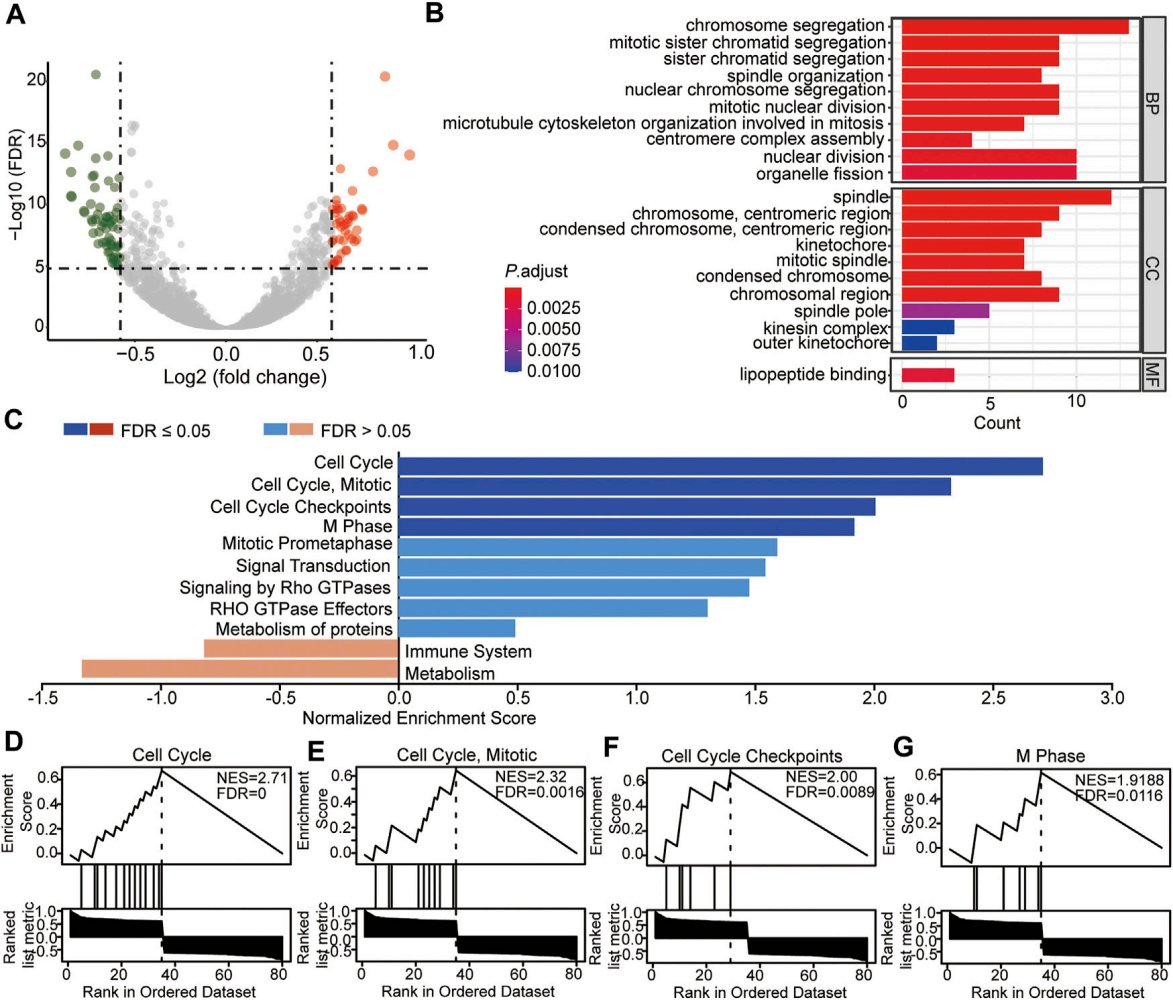
Identification and enrichment analysis of DEGs between the two risk-subtypes. **(A)** Volcano plot showing the DEGs in high-risk vs. low-risk groups. **(B)** Enriched GO terms of DEGs in the high-risk group. **(C)** GSEA analysis of DEGs in the high-risk group **(D–G)** GSEA enrichment with the threshold of FDR<0.05 of DEGs in the high-risk group. NES: normalized enrichment score.

To analyze the molecular bioprocess of the four prognostic biomarkers, DEGs in the high- vs. low-risk group were further analyzed. The volcano plot showed that with the threshold of fold change = 1.5 and *p* < 0.05, a total of 106 genes (45 genes were higher and 61 genes were lower in the high-risk group) were differentially expressed between the two groups (Figure 10A, Supplementary Table S15). The subsequent GO analysis showed that these DEGs were cell division regulation-related genes (Figure 10B). The Gene Set Enrichment Analysis (GSEA) result showed that genes enriched in the high-risk group were cell cycle regulation-regulated genes (Figures 10C–G), and genes enriched in the low-risk group were immune response- and metabolism-related genes (Figure 10C). These results were consistent with the DEGs based on the high *TEAD4* expression, suggesting that the high *TEAD4* expression affects the cell cycle, immune response, and metabolism regulation. Through this regulatory mechanism, the proliferation and invasion capacity of cancer cells were improved. Hence, *TEAD4* is a valuable biomarker for the prognostic prediction of patients with LUAD.

### The Small-Molecule Perturbagen Chemotherapeutics Forecast for High-Risk Patients

**FIGURE 11:**
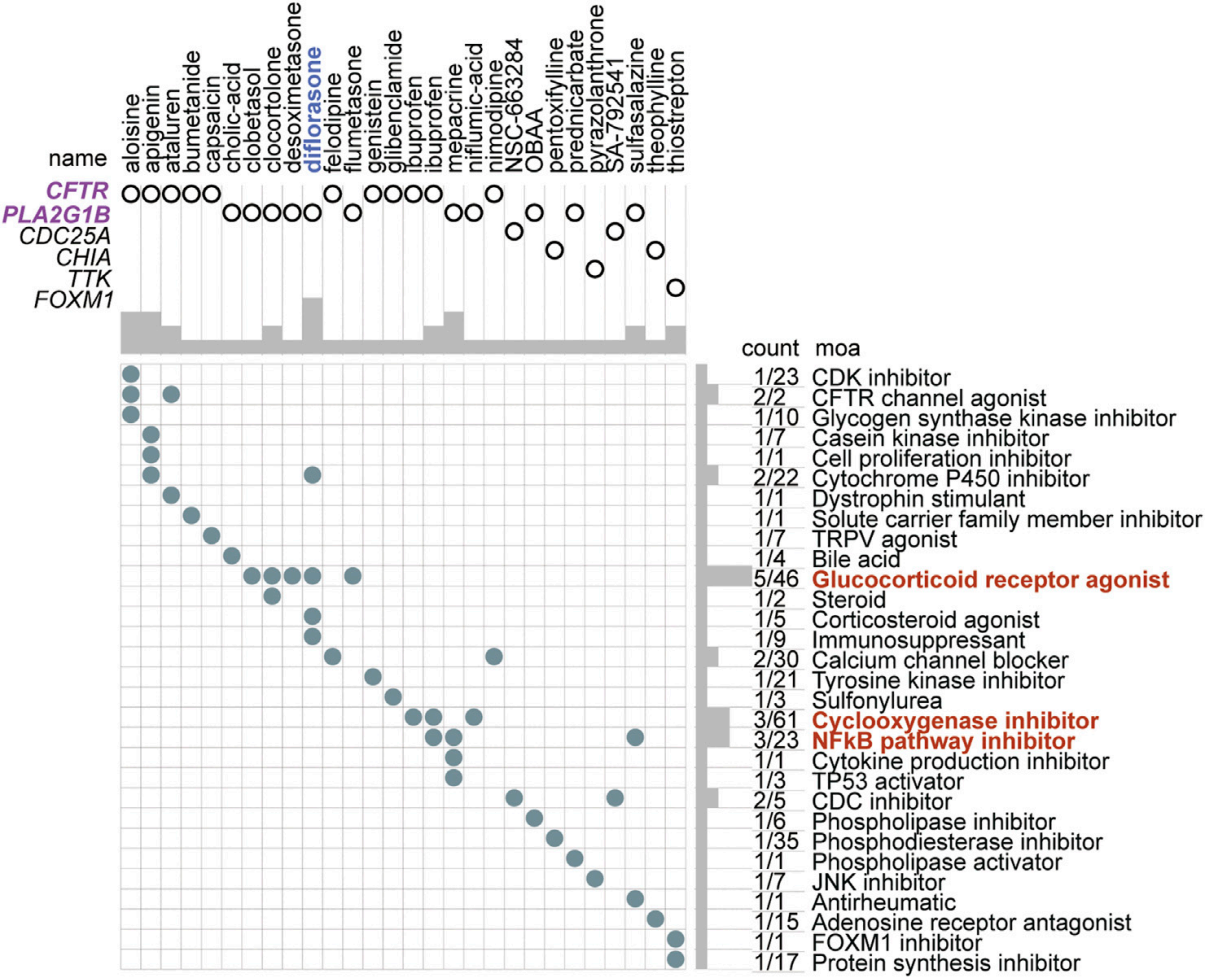
Small-molecule perturbagen chemotherapeutics forecast for high-risk patients based on the DEGs. The chemotherapeutics forecast for the high-risk patients is based on the DEGs.

According to the DEGs in the two risk subtypes, the adjuvant chemotherapeutics for high-risk patients were predicted *via* the mode of action (moa) module of the CMap database. The results showed that several small-molecule perturbagens (e.g., diflorasone, aloisine, apigenin, and mepacrine), targeting the CF transmembrane conductance regulator (*CFTR*), phospholipase A2 group IB (*PLA2G1B*), cell division cycle 25A (*CDC25A*), chitinase acidic (*CHIA*), TTK protein kinase (*TTK*), and forkhead box M1 (*FOXM1*), were the potential chemotherapeutics for the patients with a higher risk score. We also found that these potential chemotherapeutics may function by the moa of the CDK inhibitor, CFTR channel agonist, cytochrome p450 inhibitor, glucocorticoid receptor agonist, a cyclooxygenase inhibitor, and NFkB pathway inhibitor (Figure 11).

## Discussion

LUAD, one of the most frequently diagnosed cancers, is a heterogeneous disease that is commonly triggered by the alteration of key genes including oncogenes and tumor suppressors (de Sousa and Carvalho, 2018; Gavralidis and Gainor, 2020). A better understanding of the risk genes of LUAD and their specific molecular mechanism will facilitate the prevention and management of LUAD. With the rapid development of molecular biological technology and public databases, increasing numbers of biomarkers associated with the prognosis and diagnosis of LUAD have been acknowledged in recent years. However, few factors are of real clinical value.

This current study reveals an immune regulation-related biomarker, *TEAD4*, for the prognosis prediction and diagnosis of LUAD. *TEAD4* is widely studied as a Hippo signaling pathway-related transcription enhancer factor domain family gene, that interacts with YAP/TAZ to act as a transcription factor (Wu Y et al., 2021). This study first showed that the high *TEAD4* expression is associated with the prognosis in LUAD patients. Subsequent analysis determined that *TEAD4* was an independent prognostic signature in LUAD. In addition, the DEGs related to the *TEAD4* differential expression were involved in pathways of the cell cycle, immune response, and metabolism regulation. Increasing evidence suggests that the dysregulation of the immune response has been widely reported to be linked to antitumor immune escape and associated with poor outcomes in cancers (Gerada and Ryan, 2020). Dysfunction of metabolites and their regulators is emerging as a key factor affecting both cancer progression and therapeutic responses (O'Sullivan et al., 2019; Wu Q et al., 2021; Xia et al., 2021). These results all indicate that LUAD patients with different *TEAD4* levels may have distinguishing antitumor abilities and further outcomes. TEAD4 has been reported to be a protumor factor in LUAD, including its functions in promoting cancer cell proliferation, migration, and therapy resistance (Zhang et al., 2018; Gu et al., 2020; Hu et al., 2021; Yan et al., 2022). Our conclusions are consistent with these reports. However, the molecular mechanism of this gene in LUAD has not been systematically studied in previous reports. This study conducted a systematic analysis of a large number of samples that, combined with the clinical risk factors for LUAD, revealed more possible mechanisms of its pro-metastatic effects in LUAD and explained more potential reasons for the poor prognosis caused by its high expression at the macro level. This provides possible research directions for further studies on the anticancer function of this gene, such as metabolic regulation and the relationship between macrophage infiltration and *TEAD4* disorder. Our result that *TEAD4* is downregulated in LUAD seems to conflict with the prognostic result in our study, but it can be explained by the following possibility. First, during tumorigenesis, a large number of genes are changed at the expression level. The general trend is that oncogenes are activated by upregulation, while tumor suppressors are disrupted in the function (Lee and Muller, 2010). These factors all predispose the development of tumor cells. However, tumor cells live in a complex microenvironment composed of a variety of cells, including immune cells, cancer-associated fibroblasts, cancer stem cells, the extracellular matrix, and blood vessels (Arneth, 2019). Tumor growth is jointly regulated by these multiple cells and their secreted factors, and some protumor genes were not upregulated to limit their unlimited growth. Moreover, there is considerable gene expression heterogeneity in tumors among different populations. The results of RNA sequencing represent the overall expression of genes in all cancer patients but not the specific expression of genes in individuals. In summary, the regulatory role of *TEAD4* in LUAD still needs to be further explored, which is of great significance for finding novel potential therapeutic targets for LUAD.

Among the 102 DEGs in *TEAD4*-high expression subtypes, four genes including *CPS1*, *ANLN*, *RHOV*, and *KRT6A*, were identified to be independent prognostic signatures after univariate Cox regression, LASSO regression, and multivariate Cox regression. The four genes were all positively correlated with the *TEAD4* expression in LUAD, indicating that they were *TEAD4*-related signatures. The enhanced expression of this four-gene signature represents the populations of high risk. However, this was caused by the high *TEAD4* expression. In other words, the high expression of *TEAD4* led to a poor outcome partly by improving the four-gene signature expression. This study highlighted the relationship between the overall survival and immune microenvironment estimation of patients and the elevated expression of these genes and indicated that the high expression of TEAD4 predicted the poor outcome and the potential immunotherapeutic resistance by improving the four-gene signature expression. In addition, similar to *TEAD4*, three of these genes (*ANLN*, *KRT6A*, and *RHOV*) were associated with poor outcomes in LUAD, which were all recognized as oncogenic genes. For instance, *ANLN* is a well-known oncogene that promotes carcinogenesis and therapeutic resistance in multiple types of cancers, such as LUAD (Long et al., 2018; Xu et al., 2019; Deng et al., 2021), oral cancer (Wang B et al., 2021), colorectal cancer (Liu et al., 2022), breast cancer (Wang et al., 2020; Maryam and Chin, 2021), pancreatic cancer (Wang et al., 2019), and head and neck squamous cell carcinoma (Guo et al., 2021). *RHOV* has been widely studied to promote LUAD cell growth, metastasis, and therapeutic resistance (Chen H et al., 2021; Zhang et al., 2021). In addition, *KRT6A* has been shown to participate in tumor proliferation, invasion, EMT, and cancer stem cell transformation in lung cancer (Yang et al., 2020; Zhou J et al., 2021; Che et al., 2021). *CPS1* has been reported to be an oncogene that is upregulated and has prognostic significance in LUAD (Wu et al., 2020). Our study here found that *CPS1*, *ANLN*, *RHOV*, and *KRT6A* were *TEAD4*-related independent prognostic signatures in LUAD. This finding indicates that the four genes were regulated by *TEAD4* or the Hippo pathway, which provides an innovative theoretical basis for further research on the regulatory mechanism of these genes. It also provides more possibilities for studying the anticancer mechanism of these genes. Additionally, our study is the first to combine these four oncogenic genes and divide the LUAD sample into two risk subgroups according to the risk score, calculated by the combination of the expressions of the four genes and the survival of patients. Compared with previous studies on the four genes, the present study focuses more on the analysis of the common prognostic value of the four genes in combination with clinical risk factors. These four genes were innovatively identified as independent risk factors for LUAD to predict prognosis in conjunction with other clinical risk factors and provide a new theoretical basis for the choice of individual treatment for patients. Finally, through systematic analysis of data from different databases, the consistent results confirm the prognostic value of the four-gene signature and further highlight the non-negligible role of these genes in human cancer.

The TME estimation of the two risk subtypes suggests that the high-risk group has higher stromal, immune, and ESTIMATE scores, as well as a lower score of tumor purity. The proportions of plasma cells, resting memory CD4 T cells, monocytes, resting dendritic cells, and resting mast cells were lower in the high-risk group, while the proportions of CD8 T cells, activated memory CD4 T cells, resting NK cells, M0 macrophages, M1 macrophages, activated dendritic cells, activated mast cells, and neutrophils were higher. Tumor-associated macrophages (TAMs) are among the most abundant immune cells in the TME and act to enhance tumor progression and metastasis (Mantovani et al., 2017; Cassetta and Pollard, 2018; Lopez-Yrigoyen et al., 2021). High infiltration of TAMs is associated with poor prognosis in several types of cancer, such as breast cancer, ovarian cancer, bladder cancer, and NSCLC (Yin et al., 2017; Zhao et al., 2017; Kowal et al., 2019; Lopez-Yrigoyen et al., 2021). The microenvironmental stimuli and signals that encounter each specific tissue always induce macrophage polarization. According to the specific inducers, two major macrophage subpopulations, classically activated or inflammatory (M1) and alternatively activated or anti-inflammatory (M2) macrophages, have been identified (M0 macrophages are naïve macrophages without polarization) (Miao et al., 2017; Huang et al., 2019; Kang et al., 2020). Functionally, M1 macrophages have robust antimicrobial and antitumoral activity, by removing pathogens during infection (Shapouri-Moghaddam et al., 2018), while M2 macrophages participate in angiogenesis, immunoregulation, tumor formation, and progression (Shapouri-Moghaddam et al., 2018). The different levels of infiltration of immune cells directly determine the different prognoses of patients.

As another indicator of immune escape, MHC-I and MHC-II molecules were found to be lower in the high-risk subtype than in the low-risk group. Degrading MHC-I is always a cause of immune evasion, a major obstacle for cancer therapy, which has been implicated in resistance to immune checkpoint blockade (ICB) therapy (McGranahan et al., 2017; Rodig et al., 2018; Burr et al., 2019; Yamamoto et al., 2020a; Yamamoto et al., 2020b; Zhou Y et al., 2021; Dhatchinamoorthy et al., 2021). During the process of immune evasion, MHC-I downregulation is one major mechanism to avoid antitumor immunity by reducing recognition by cytotoxic CD8^+^ T cells, often correlating with poor prognosis (Cornel et al., 2020). In addition to MHC-I and MHC-II, an antigen-presenting complex traditionally associated with professional antigen-presenting cells (APCs) is critical in antitumor immunity (Axelrod et al., 2019). Tumor-specific MHC-II is reported to be associated with superior prognosis, allowing recognition of tumor cells by the immune system, thus playing a role in immunotherapy and improving the response to ICB therapy (Mortara et al., 2006; Forero et al., 2016; Johnson et al., 2016). The downregulated MHC-I and MHC-II molecules in the high-risk subtypes indicate the potential strong immune escape and ICB therapy resistance of the patients in this group.

Moreover, immune checkpoint genes were also detected, and the results showed that *CD274* (*PD-L1*) and *LAG3* (CD223) were highly expressed in the high-risk subtype. PD-L1 (Programmed death-ligand 1) is expressed on several types of tumor cells, mediating the tumor-induced immune suppression (immune checkpoint) by binding with the receptor PD-1 (programmed cell death protein 1), which is highly expressed in activated T cells, B cells, dendritic cells, and natural killer cells (Dermani et al., 2019). The binding of PD-L1 to PD-1 on T cells results in the inhibition of cancer cells by destruction by T cells, thus promoting immune escape (Gou et al., 2020). Therefore, PD-L1 or PD-1 monoclonal antibodies have been used for cancer treatment (Bagchi et al., 2021; Carlino et al., 2021; Doroshow et al., 2021). A higher level of PD-L1 predicts a worse outcome in patients. In addition, *LAG3* (lymphocyte activation gene 3, CD223) is another kind of inhibitory receptor (IRs) that has been reported to play a negative regulatory role in cancer immunology by interacting with its ligands (Wang M et al., 2021). *LAG3* expression is also shown to be positively associated with *CD274* (*PD-L1*) (Wang M et al., 2021). TEAD4 is known as a transcription factor associated with resistance to different therapeutic approaches (Jiao et al., 2017; Yu et al., 2021; Yan et al., 2022). Unfortunately, these studies were not confirmed in patients with LUAD. Our study systematically analyzed the TME in patients with LUAD and demonstrated that a high expression of *TEAD4* is associated with a poor anti-tumor immune response, with evidence of a lower immune score and HLA family components and higher levels of immune checkpoint genes in the high-risk subgroups based on the high *TEAD4* expression. This not only reinforces previous research but also provides new insights into the mechanisms of this gene involved in therapy resistance. Finally, DEGs between the two risk groups were identified, and the subsequent GO and GSEA showed that cell division and cell cycle regulation-related genes were enriched in the high-risk group, while immune response- and metabolism-related genes were enriched in the low-risk group. This is consistent with *TEAD4*-high expression-related DEGs, as well as the TME result in the Cox model, further confirming the conclusion in this study.

According to these DEGs, the forecasted adjuvant small-molecule drugs for the high-risk subtype are perturbations targeting *CFTR*, *PLA2G1B*, *CDC25A*, *CHIA*, *TTK*, and *OXM1* by the moa of the CDK inhibitor, CFTR channel agonist, cytochrome p450 inhibitor, glucocorticoid receptor agonist, cyclooxygenase inhibitor, or NFkB pathway inhibitor. Targeting these pathways may be an efficient therapeutic strategy for patients with high levels of *TEAD4*. A total of 28 potential small-molecule drugs were predicted based on the specific differentially expressed genes in these populations. This not only provided a novel solution to the low survival of patients with LUAD but also laid a theoretical foundation for further drug research and development.

However, there are several limitations to the present study that should be stated. First, despite the fact that bioinformatic technology is powerful in efficiently understanding biological functions, the underlying mechanisms of these genes in LUAD still need further cellular explorations. Moreover, clinical tissues and paired adjacent normal tissues should be collected to further detect the protein expression level of TEAD4, as well as the related cell cycle and immune response genes in LUAD. Second, as this is a retrospective study, missing data and selection biases were inevitable, and the statistical power might be low. Therefore, further studies with a large sample size are warranted to increase the statistical power. Finally, due to lack of data about immunotherapies, the relationship between the *TEAD4* expression and ICB therapy response cannot be investigated. More clinical and demographic characteristics of LUAD patients need to be included in further studies.

In summary, our results suggest that *TEAD4* is a novel molecular biomarker for diagnosis and prognosis, predicting overall survival and immune microenvironment estimation in LUAD. However, large prospective studies are warranted, and further experimental validation should be performed to prove the prognostic value of this gene in LUAD.

## Data Availability

Publicly available datasets were analyzed in this study. These data can be found at: https://xenabrowser.net/datapages/?cohort=GDC%20TCGA%20Lung%20Adenocarcinoma%20(LUAD)&removeHub=https%3A%2F%2Fxena.treehouse.gi.ucsc.edu%3A443.

## Supplementary Material

Supplementary Figure S1 Heatmap of DEGs in *TEAD4*-high vs. *TEAD4*-low groups.

Supplementary Figure S2 Validation of the Cox model with GEO data. **(A–C)** Overall survival of high- and low-risk groups, respectively, in GEO validation set#1 **(A)**, validation set#2 **(B)**, and validation set#3 **(C)**. **(D–F)** ROC analyses of the model for 1, 3, and 5 years, respectively, in GEO validation set#1 **(D)**, validation set#2 **(E)**, and validation set#3 **(F)**.
