# Supplementary figures and images for: A Four-Gene Prognostic Signature Based on the TEAD4 Differential Expression Predicts Overall Survival and Immune Microenvironment Estimation in Lung Adenocarcinoma
Source: Front Pharmacol. 2022 May 4;13:874780. doi: 10.3389/fphar.2022.874780 (PMC9114646; doi:10.3389/fphar.2022.874780)

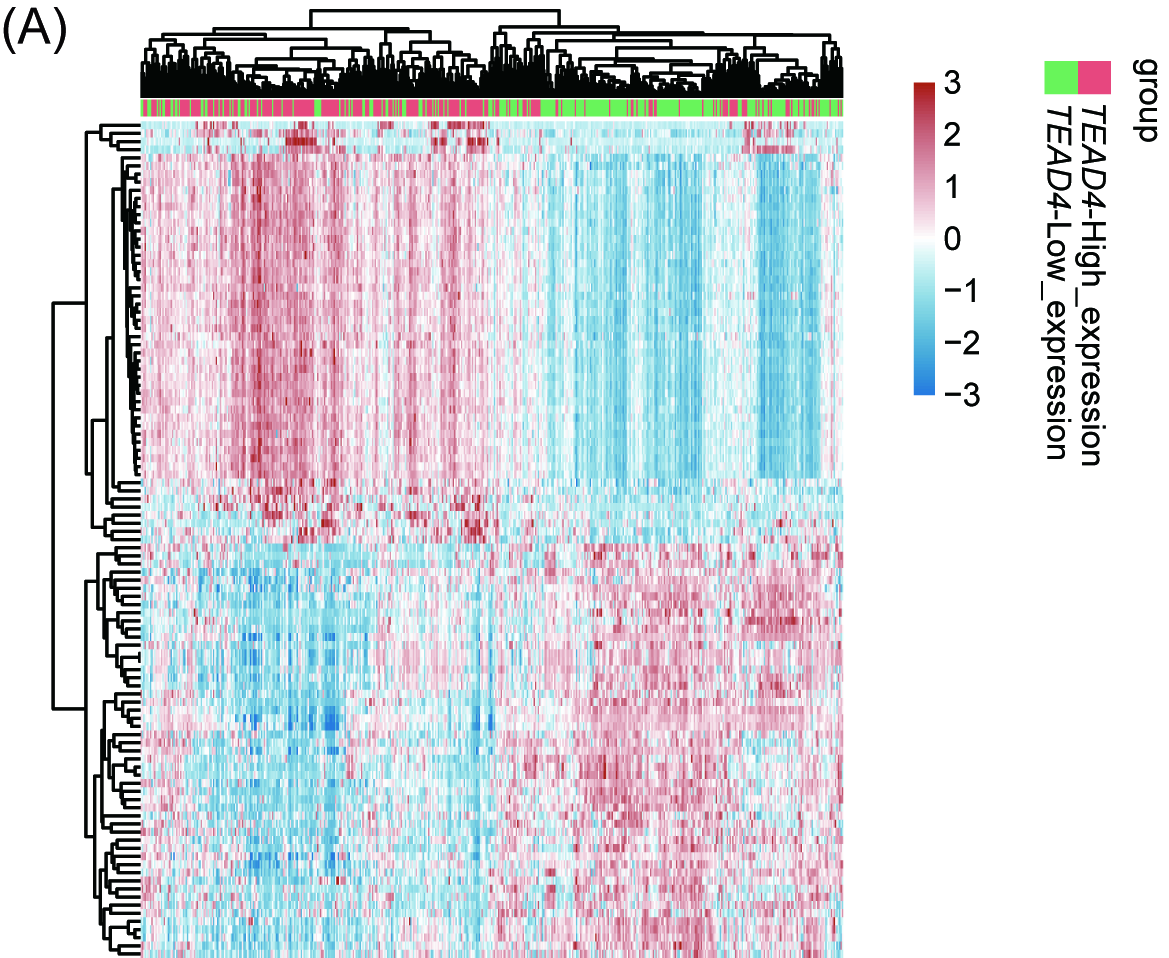

Supplement: Supplementary file 8 [file Image1.tif]

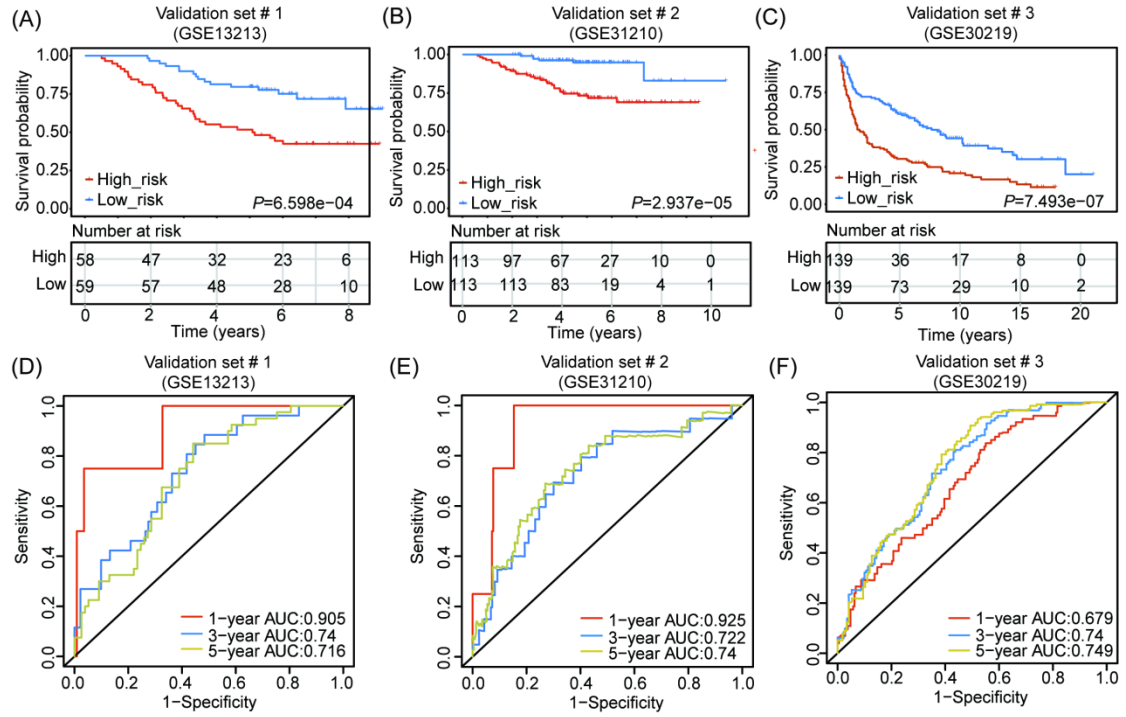

Supplement: Supplementary file 9 [file Image2.pdf]
